# An Overview of Recycling Phenolic Resin

**DOI:** 10.3390/polym16091255

**Published:** 2024-04-30

**Authors:** Bing Zhu, Xinyao Jiang, Songjun Li, Maiyong Zhu

**Affiliations:** School of Materials Science & Engineering, Jiangsu University, Zhenjiang 212013, China

**Keywords:** phenolic resin, mechanical recovery, chemical recycling, carbon material

## Abstract

Over a century ago, phenolic formaldehyde (PF) resin was developed and continues to increase in yield due to its diverse applications. However, PF resin is a thermosetting plastic lacking fluidity and moldability, which are nondegradable in natural environments, leading to severe threats to fossil resources as well as global environmental crises. As a result, recycling PF resin is extremely important. In this review, we provide the recent advances in the recycling of PF resin, which includes mechanical recycling, chemical recycling, and utilization of carbon-based materials. The advantages and disadvantages of each strategy are evaluated from a green chemistry perspective. This article aims to attract interest in PF resin design, synthesizing, application and recycling, offering useful suggestions.

## 1. Introduction

**Figure 1:**
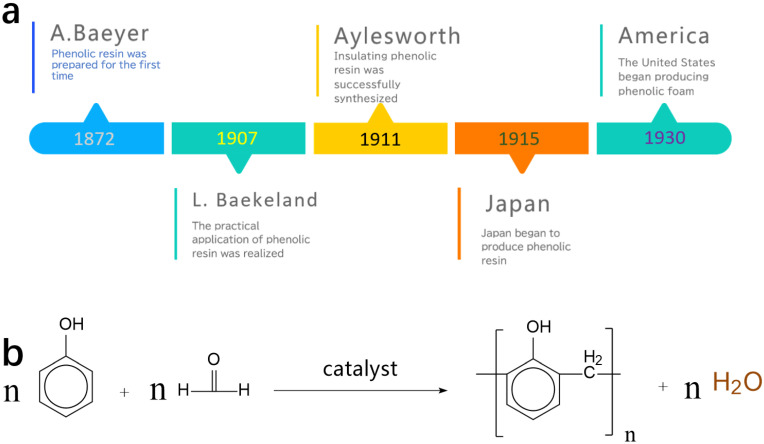
(**a**) Early development of phenolic resin; (**b**) simple synthesis equation of phenolic resin.

Phenolic resin, also known as phenol formaldehyde resin (PF), is the first synthetic polymer material, dating back to 1872. Bayer discovered phenolic resin when phenol reacts with aldehyde. Although some chemists invented PF, they did not apply it or abandon it due to its difficulty in handling. Until 1907, Leo Baekeland [1,2] was convinced that this novel substance had many excellent physical and chemical properties, like being non-flammable, inert to acid and alkali, lightweight, together with high hardness. In a sense, Leo Baekeland was the first person who discovered phenolic resin. He made large amounts of contributions to the field of phenolic resin in his lifeline. Figure 1a show a timeline of the development of phenolic resin from its first synthesis to its worldwide application. Figure 1b shows a simple equation for the synthesis of phenolic resin. Like general polymer compounds, the phenolic resin has the basic characteristics of polymer compounds, such as high molecular weight, molecular structure diversity, curing properties and pyrolysis charring properties. In addition, due to its unique chemical structure and macromolecular cross-networking structure, it has excellent adhesion, heat resistance, and ablative resistance and is flame retardant. Therefore, phenolic resin has been widely used in the fields of molding plastics, molding materials, thermal insulation materials, sound insulation materials, coatings, packaging materials and refractory materials.

### 1.1. The Consumption of Phenolic Resin in the Global Market

**Figure 2:**
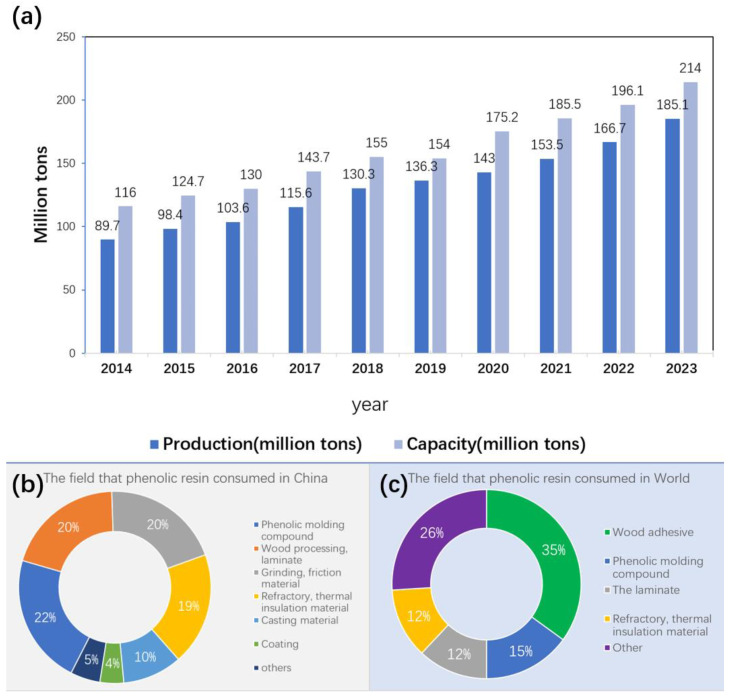
(**a**) Production and capacity of phenolic resin in China in 2014–2020; (**b**) the field that phenolic resin consumed in China; (**c**) the field that phenolic resin consumed in the world. Reproduced with permission from ref. [3]. Copyright 2019 published under a CC-BY license.

With the development of the industry, phenolic resin has been applied in diverse production of products and construction due to its outstanding performance. Industries involved include but are not limited to the automobile and aerospace industries in terms of the use of composite materials. According to the data in Figure 2a, the production of phenolic resin in China is increasing year by year. Until 2020, China’s production of phenolic resin was about 1.43 million tons, and the phenolic resin production capacity was about 1.752 million tons. It can be seen that the production scale of phenolic resin is still large and continues to increase. The demand for phenolic resin is predicted to rise steadily over the next three years, with an annual compound growth rate of consumption of 5.3%, due to the ongoing expansion of the phenolic resin industry scale, advancements in production technology, and improved product quality. In 2025, the consumption of phenolic resin is estimated to reach 1.85 million tons.

Before 2017, China’s import of phenolic resin was higher than its export, indicating its wide application market. The material is primarily used in phenolic molding compounds, wood processing, and refractory and thermal insulation materials (Figure 2b) [3]. These fields are heavily reliant on the outstanding properties of phenolic resin. As we look globally, the production and application of phenolic resin will continue to amaze people. In 1945, just a year after Baekeland’s death, the United States produced more than 400 thousand tons of phenolic resin a year. After the 1940s, the synthesis method of phenolic resin tended to be mature and began to be diversified. In the 1970s, many thermosetting and thermoplastic resins appeared, such as vinyl resin, epoxy resin [4], polypropylene, ABS, etc. After the 1980s, with the improvement of the transportation industry and the prosperity of the construction industry, the demand for phenolic resin increased significantly. Therefore, the development trend of phenolic resin was still great. Even if COVID-19 affects the business landscape, the global phenolic resin market is estimated at $13 billion in 2020 and is expected to reach $17.9 billion by 2027, with an annual growth rate of nearly 5%. The phenolic resin market in the Americas is estimated to be $3.5 billion in 2021 and is expected to reach $3.68 billion in 2022, with a CAGR of 6.16%, as well as an expectation to reach $5 billion in 2027. The main fields that phenolic resin used in the world are shown in Figure 2c.

### 1.2. Environmental Hazards of Phenolic Resins

The synthesis of PF resin typically involves adding formaldehyde to the ortho and para positions of the phenol. It is well known that the benzene ring can be activated by electron-donor groups, such as -NH_2_, -OH and so on, by increasing the electron density of the benzene ring. Meanwhile, the addition reaction usually occurs at both the ortho and para positions of -OH in the benzene ring. As a consequence, in this step, a mixture containing mono-, di- or tri-hydroxymethylphenol intermediates is generated. The second step refers to the condensation of resulting monomers, forming methylene bridges. In view of the operation, the formation of PF resin can be achieved either by an acid catalytic system or a base catalytic system.

**Figure 3:**
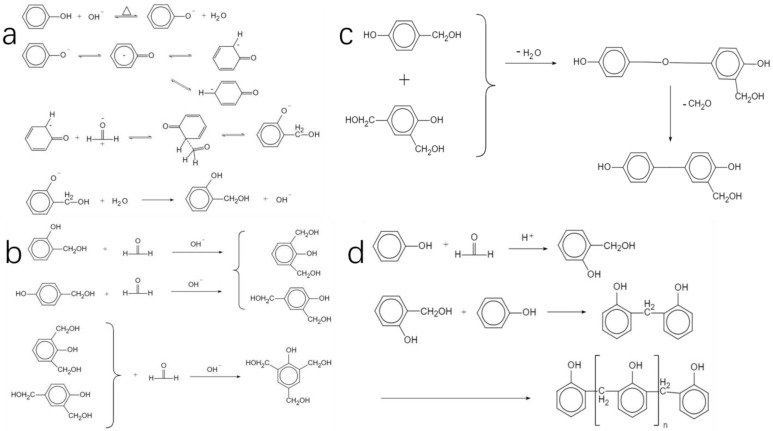
(**a**) Formation process of phenol-hydroxymethyl; (**b**) addition reaction; (**c**) polycondensation reaction; (**d**) synthesis of thermoplastic phenolic resin; reproduced with permission from ref. [3]. Copyright 2019 published under a CC-BY license.

(1) Alkaline system: The synthesis equations of phenolic resins in alkaline systems are shown in Figure 3. Under the alkaline condition, phenol reacts with OH- in the solution to form phenoxy anion and the electrons on the oxygen ions in the phenoxy anion form an extended pi bond with the benzene ring. Due to the electron-withdrawing effect of oxygen ions, the electron density of the ortho and para positions and nucleophilicity of the whole structure increase, which leads to the addition reaction mainly concentrated on the ortho and para positions of phenol. In formaldehyde, the electronegativity of the oxygen atom is larger than that of the carbon atom, resulting in a greater attraction of the oxygen atom to the valence electrons showing negative electricity, and positive electricity in the carbon atom. Formaldehyde reacts with the active center to produce ortho- or para- hydroxymethyl-phenol (Figure 3a). The hydroxymethyl-phenol can continue to react with formaldehyde to form di-hydroxymethyl-phenol and tri-hydroxymethyl-phenol [5] (Figure 3b). The process of the formation of methylene bridges is shown in Figure 3c. Since addition reactions can occur in the ortho position or para positions, there are many types of chemical bonds between dimers and oligomers.
(2) Acid system: It is easy to form linear phenolic resin under acidic conditions because the condensation rate is much higher than the addition rate under acidic conditions. The specific chemical reaction equations are shown in Figure 3d.

Phenolic resin, despite its different synthesis mechanisms under acidic and alkaline conditions, is not environmentally friendly. It can volatilize toxic substances at high temperatures, with high levels of free phenol and formaldehyde, which are carcinogenic and pose significant harm to the environment and organisms [6]. The biggest hazard of phenolic resin is these free formaldehyde and phenol. Formaldehyde is a carcinogen, and studies have found that long-term exposure to formaldehyde can lead to an increased risk of cancer [7]. The United States National Toxicology Program (US-NTP) and other research institutions all have classified formaldehyde as a weak genotoxic and probable carcinogenic for humans [8]. Biological experiments have also proved that long-term exposure to formaldehyde can cause damage to the central nervous system and immune system [9]. The harm of inhaling a small amount of formaldehyde to the nervous system is mainly manifested as headache, insomnia, dizziness and other adverse reactions. Long-term exposure to formaldehyde can cause neurological diseases, brain cancer, and even memory impairment [8]. Formaldehyde has a strong irritation; when the concentration is low, people’s eyes have a spicy feeling, tears and red swelling. When the concentration is slightly higher, people will feel choking, nausea and even breathing difficulties and suffocation. There are a large number of hydroxyl, sulfhydryl and amino groups in the cell and enzyme system of the human body, which will react immediately after contact with formaldehyde, resulting in cytotoxicity and denaturation of proteins. Acute formaldehyde poisoning will not only cause respiratory discomfort but also harmful effects on human metabolism, which may lead to renal failure. Long-term exposure to formaldehyde not only worsens these diseases but may also lead to death [10].

Phenol is a colorless liquid with a characteristic odor that is soluble in water and organic solvents. Considering the toxicity and environmental pollution of phenol, many countries have controlled the production and use of phenol. Phenol has been inscribed into the Priority Pollutant List by the US Environmental Protection Agency (US EPA) [11]. In China, phenol is one of the substances on the list of priority pollutants held by the China Environmental Priority Monitoring Research Group. At the same time, the International Maritime Organization (IMO) also rated phenol as one of the top 20 chemicals with the highest risk of causing hazardous and noxious incidents [12]. J. Michałowicz et al. pointed out that the essential factor in evaluating the toxicity of phenol is the reaction ability between phenol and biological cells [13]. The effects of acute phenol poisoning on the human body are varied. Phenol can stimulate skin cells on the human surface and lead to skin necrosis because phenol reacts with aminoacids contained in epidermal keratin and endothelial collagen [14]. Experiments have shown that even skin contact and absorption of phenol solution can lead to biological death. Chronic administration of phenol in animals can lead to pathological changes in biological tissues, while prolonged exposure to phenol in humans can lead to anorexia, weakness, muscle atrophy, headache and other symptoms [15]. In addition to the effects on animals and humans, the harm of phenol to the soil ecosystem cannot be ignored. Chae et al. conducted a large number of experiments on previous studies, providing strong data support for the conclusion that phenol can cause harm to soil ecosystems [16]. Similarly, the risks of phenol to aquatic organisms and ecosystems have been revealed [12]. Api et al. [17] have summarized and discussed all the toxicity of phenol, and will not go into too much detail here.

Solving or avoiding the production of these free toxic substances has become an important aspect of green production and use of phenolic resin. Phenolic resin waste, like other plastic waste, has a significant impact on the environment, including visual pollution, land pollution and ecological damage. Phenolic resin, as a thermosetting resin, is not as easy to handle as thermoplastic resin. Many thermoplastic resins have very extensive recycling methods, such as PVC [18], PET [19] and PE [20,21]. There is also a lot of literature on the recycling of some thermosetting resins, such as epoxy resins [22]. In general, the recycling of thermosets is generally more difficult than thermoplastics and is relatively less researched.

**Figure 4:**
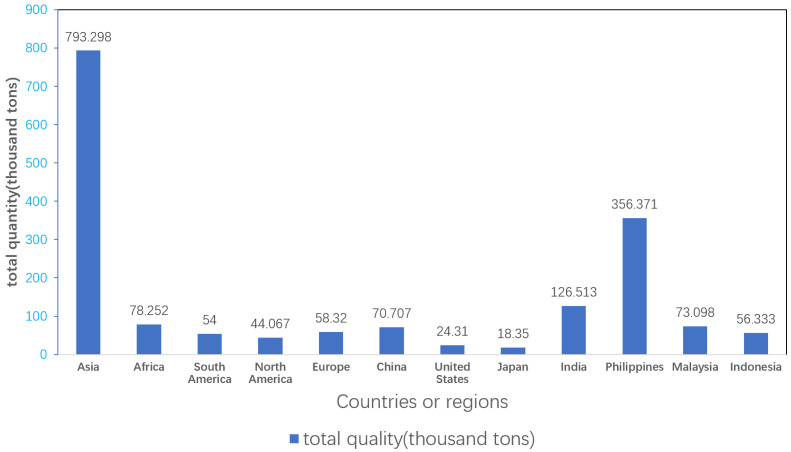
Plastic waste emitted to the ocean.

The most severe damage is to wildlife, as plastic products can hinder their normal activities, entangle many creatures in the ocean, and cause harm to sea turtles, seals, whales and seabirds. As Figure 4 shows, various countries and regions are releasing large amounts of plastic into the ocean. Additionally, unintentional plastic ingestion can lead to adverse health consequences, such as false feelings of fullness, blockage of the intestine, stomach rupture, metabolic disorders, biological enzyme activity changes and cell necrosis. The harmful effects of plastic waste are a significant concern for the health of our planet. When microplastics are further broken down into smaller particles, they are likely to be absorbed by the body’s circulatory system and enter the body’s organs. In addition, these plastics may have chemicals in the manufacturing process that can harm or even kill cells. Toxic chemicals leach from plastic and come into contact with the human body through drinking water, which can potentially cause cancer, birth defects, compromised immunity or other diseases. The harm of plastic is omnipresent, plastic is not degradable and it persists in the environment and synthetic physiological fluids.

As the simplest way to dispose of plastic waste, a landfill has many limitations. Although there is a multitude of literature discussing how to rationalize landfills [23], even in developed countries, landfills are poorly managed, and many are left in open landfills or dumped into the ocean. In addition to management problems, landfills also bring greenhouse gas emissions and leachate pollution problems. Pyrolysis [24] is also widely used to degrade waste phenolic resins. Jackson [25], Parker and Ouchi put forward and illustrate three mechanisms of thermal degradation. Mong et al. [26] compared the advantages and disadvantages of different types of pyrolysis. Most pyrolysis processes require a high temperature (300–850 °C) and emit COx, SOx and NOx in the pyrolysis process [27]. Although mechanical recycling is also a good solution to the waste of many plastics, it also has its shortcomings. Thermal decomposition or mechanical damage in the processing process will reduce the quality of the material [28,29]. The practical value of the material after continuous mechanical recycling may be very low, which belongs to a kind of degraded recycling.

This paper describes and summarizes how to achieve more green and sustainable goals in the recycling aspect of phenolic resins. In this paper, the recycling chain is divided into two sections: mechanical and chemical recycling. In mechanical recycling, how to optimize the performance of mechanical recycling products becomes the focus of discussion, and the feasibility of preparing simple composite functional materials from recycled products is also described. The chemical recovery section is categorized and discussed based on the differences between the three recovery methods. The depolymerization section focuses on methods that break the chemical bonds of phenolic resins. A novel method was discovered for converting phenolic resins into carbon materials, involving the conversion of phenolic resin waste into aromatic amines. In the section on mechanical recycling, the novelty is found in that mechanical recycling does not lead to a wide range of mechanical properties as everyone thinks, but, through reasonable adjustments to the formula, it can be carried out to improve some of the performance, and even the overall mechanical properties of the product will not be reduced. In the section on chemical recycling, instead of just discussing how to turn phenolic resin polymers into monomers, a new method of converting phenolic resins is proposed and two specific methods are given.

## 2. Mechanical Recycling

**Figure 5:**
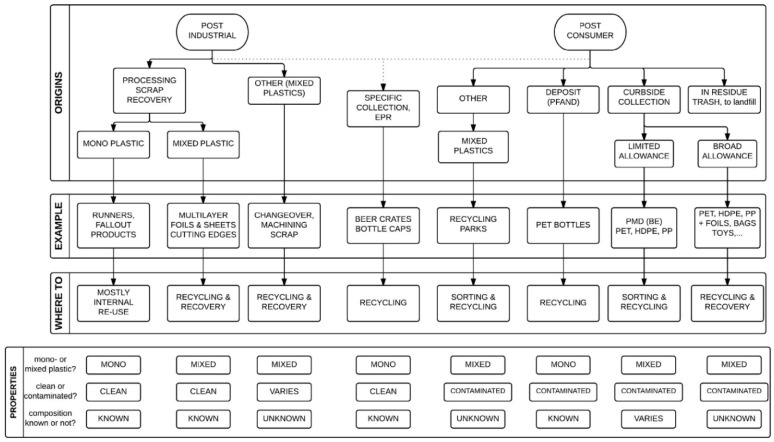
Some examples of mechanical recycling and the process of mechanical recycling. Reproduced from ref. [31] with permission. Copyright 2017 Elsevier Ltd.

Mechanical recycling is now one of the most widely used methods for recycling phenolic resin waste, which was previously disposed of in landfills. Landfills are becoming obsolete due to their increasing costs [30] and direct harm to the natural environment, as well as the growing awareness of the importance of living in harmony with nature. Gradually, mechanical recycling has become the main method of recycling. Mechanical recycling is suitable for the recycling of almost all plastics. Contrary to what most people think, mechanical recycling actually involves a series of processes from collecting, sorting, cleaning, and grinding [31]. And these processing sequences and processing times are not fixed, but change according to the type of recycled material. Figure 5 shows the mechanical recovery process with some examples. In the section on mechanical recycling, four representative mechanical recycling methods are mainly described, and the characteristics of recycling methods and the advantages and disadvantages of product performance are evaluated.

### 2.1. Mechanical Grinding and Reincorporation

Since the 1990s, mechanical grinding and reincorporation have become a method to recycle thermosetting resin [32]. This technology is mainly used in the replacement of filler fraction with powder obtained by grinding waste sheet molding compounds (SMC) or dough molding compounds (DMC). There is a large body of literature on the mechanical recovery of solid plastic wastes. However, experiments have shown that the properties of regenerated materials are reduced due to fiber damage and weak bonding between waste particles and new resins [28]. In many past experiments, the final composite always showed a reduction in mechanical properties. Although researchers changed the proportion of ingredients from time to time, the result is still less than satisfactory. Nevertheless, there is always an exception to everything. Bledzki et al. [33] first attempted to use recyclate materials substitute for the reinforcing component in DMC, and through many experiments, they drew a conclusion that the mechanical properties could be promoted.

**Figure 6:**
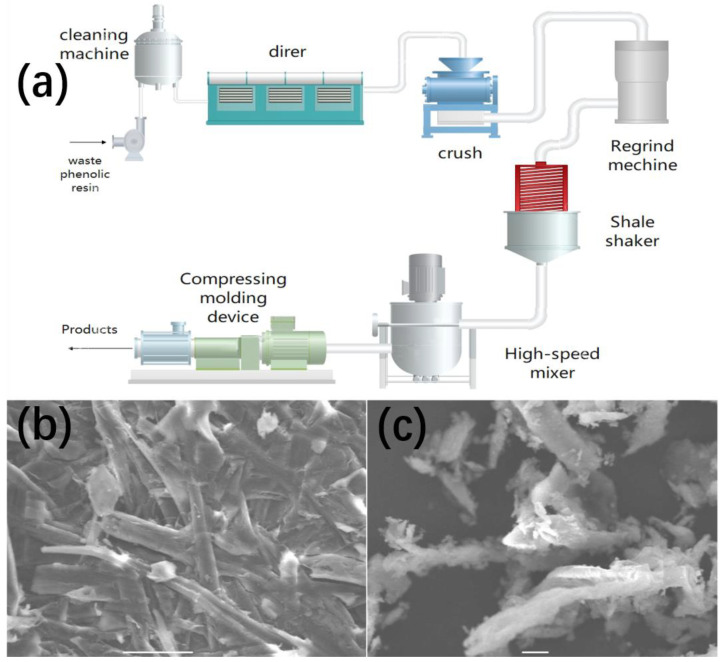
(**a**) Grinding and reprocessing processes; (**b**) SEM image of phenolic resin before regrinding; (**c**) SEM image of phenolic resin after regrinding; reprinted with permission from ref. [35]. Copyright © 2020 Jian Hu et al., published under a CC-BY license.

Waste plastics need to be ground before being used for new product synthesis. Grinding is crucial for mechanical recycling, as the size and interval of the powder affect the material’s physical properties. Experiments have shown that breaking the cross-linked bonds of phenolic resin could make it easier to recycle, as it is difficult to synthesize new products directly from waste plastics. Liu and Shi discovered a mechanical method based on this idea [34], by high-speed grinding for a long time, together with increasing shear stress and friction. Uneven stress or energy is concentrated on part of the molecular chain, and some of the chemical bonds are broken due to the accumulation of mechanical energy, while heat energy also breaks some of the weaker chemical bonds. In 2020, Hu et al also used a similar method for mechanical recovery of phenolic resin, and obtained the surface topography of phenolic resin before and after recovery [35] (Figure 6b,c). The entire recycling process is shown in Figure 6a. First, drying and cleaning the waste phenolic resin, before feeding the waste into a high-speed grinding mechanical, roughing machine should be used to cut the size of the waste, making them easier to grind. After grinding, the material falls into the shale shaker for further processing, which includes shear, extrusion, friction and others. Through this complex processing, the waste has become powder. Putting this power into a high-speed mixer, by high-speed mixing, the powder is finally pressed into some shape molds. Thus, they recycle the waste phenolic resin successfully. Speed, time, feeding material weight and feeding particle sizes are considered the main factors that affect the result of grinding. After conducting experiments many times, the author drew a conclusion that the speed in an experiment is the most important, with the size second, and weight last. Liu et al. [34] studied the effect of grinding on the grinding product in detail, but unfortunately did not specify the specific properties of the recycled material.

Bernardeau et al. also discovered a new mechanical method, mixing phenolic molding compound (PMC) wastes with thermoplastic material, such as polypropylene (PP) [36]. Mechanical grinding and reincorporation of SMC will cause a reduction in mechanical property under most circumstances, but the reduction will not happen in this method on the whole. After mixing, the composite material is found to increase in thermo-oxidative stability and mechanical properties. In contrast, in an experiment mixing the virgin phenolic resin into PP, the result indicated that the performance of recycled material could be equal to the new resin material. The entire recycling process is simple and easy to operate. The phenolic particles were dried together with polypropylene and formed by a twin-screw extruder. The PMC-filled PP was obtained after washing and drying.

As we all know, PP is a semi-crystalline polymer, which means that the degree of crystalline influence the properties of PP. In terms of the addition of PMC fillers, the degree of crystalline bond is changing. When the particle diameter is not large, the addition of a small proportion of PMC will not have a great impact on the crystallinity of PP. This phenomenon may be attributed to the polymer chain movement blocked by the filler, resulting in a polymer chain that cannot form crystallization [37]. When changing the type of fillers, it seems that the change of crystalline is random and not obvious. Except for crystalline, the particle size also has an influence on tensile and impact strength. From the results of this research, it can be seen that the PMC-filled PP sample with the highest Young modulus is up to 2.4 GPa. The Young modulus of phenolic resin is between 1.96 and 2.94 GPa. However, in terms of tensile strength, the tensile strength of the PMC-filled PP (20–35 MPa) is not as good as that of phenolic resin (60–80 MPa). In PMC-filled PP, the tensile strength decreases with the increase in particle size. When investigating the reason for the decrease in tensile strength, the low adhesion is considered to be the main factor. Although the tensile strength of the new material is not qualified enough, Young’s modulus is comparable to that of phenolic resin, and it can be seen that the product obtained by this method can partially replace phenolic resin.

**Figure 7:**
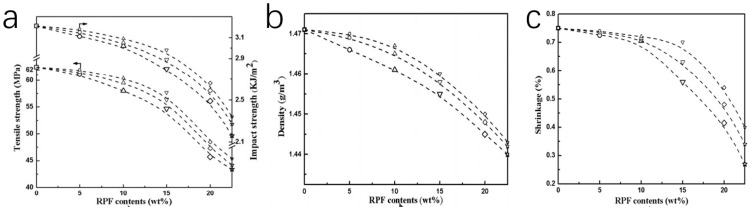
(**a**) Tensile strength and impact strength of recycled PF/RPF materials; (**b**) the density of regenerated PF/RPF materials; (**c**) the shrinkage of regenerated PF/RPF materials. Reproduced from ref. [38] with permission. Copyright 2019 Wiley Periodicals, Inc.

From these experiments, we can see that the particle size of the powder after grinding will affect the properties of the composite materials. Yang et al. investigated the effects of powders of different micron sizes on the mechanical strength of recycled PF/RPF/TOX materials [38] in depth. They studied 87 kinds of composite materials with different formulations and PF particle sizes. Fifteen of them had no added crosslinkers (trioxymethylene). As Figure 7a–c show, with the increase in PF particle concentration or particle size, the tensile strength, impact strength density and shrinkage percentage of the composite decrease. (The three shapes in Figure 7 represent three different formulations of PF composite materials. The arrow points to the kind of property that corresponds to the curve). In Figure 7a, the tensile strength and impact strength of all samples are close to each other, and there is no significant decrease compared with phenolic resin. When the content of RPF is 5 wt%, the tensile strength (60 MPa) and impact strength (2.9 KJ/m^2^) were very close to the properties of phenolic resin. This is just the case without the addition of crosslinkers. Yang et al. [38] discussed the effect of subtle changes in each component of the formulation on the final composite material and carried out very detailed experiments and tests on 87 formulations, which are not described here. Their work is important for those who want to understand the effects of recycled material formulations on material properties in mechanical recycling.

### 2.2. Mechanical Recycling and Special Applications

Although the traditional mechanical recycling method recovers the phenolic resin, the waste is reused to reduce the pollution to the environment, but these are very simple recycling materials. Different from traditional mechanical recycling methods that waste PF powder or fillers are mixed with thermosetting material or used as construction material, this method takes full advantage of the character of phenolic resin. Inventing a new flame-retardant panel by mixing the PF powder with wood fibers (WB) [39]. WB is widely used in the industry currently because of its great characteristics, such as being natural, recyclable, biodegradable and versatile [40]. As a material, WB is low-cost but inflammable. On the contrary, phenolic resin usually contains a large number of blocked phenolic sites, and a flame retardant carbonizing agent and synergist are added to its synthesis process, which ensures relatively high temperature resistance of the material [41,42]. Thus, it is both practically necessary and economically desirable to produce a new composite material by mixing WB with non-flammable recycling PF powder. However, the problem that puzzles people is their poor compatibility, as WB is polar, hydrophilic and flammable, and PF is non-polar and hydrophobic. In order to increase the adhesive capacity, we could change the ratio of two materials or add a catalyst and coupling reagent [43,44].

**Figure 8:**
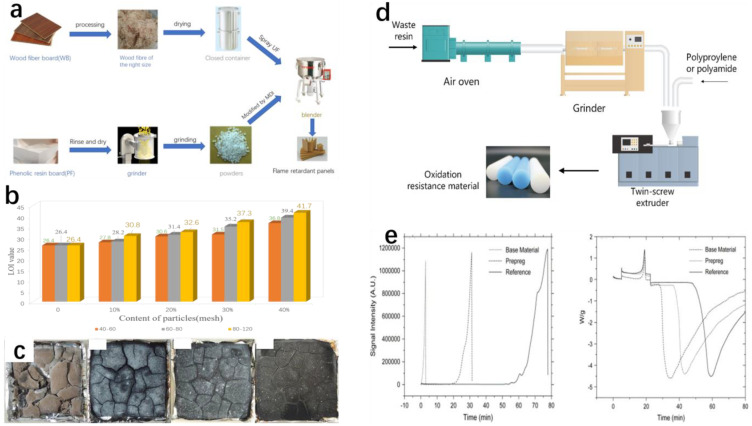
(**a**) Steps for recovery of phenolic resin and wood fiber and the production of their composites; (**b**) the influence of different particle content on LOI value; (**c**) residual carbon of WB-PF composites with different addition levels of 80–120 mesh particles (0%, 10%, 20%, 30%). Reproduced with permission from ref. [39]. Copyright 2018 Elsevier. (**d**) The preparation process diagram of the antioxidant material; (**e**) CL trace of PP composites and DSC trace of PP composites. Reproduced with permission from ref. [48]. Copyright 2005 Elsevier.

The entire recycling process is shown in Figure 8a, with reference to the production of retardant urea formaldehyde medium-density fiberboard [45], which modifies WB with urea–formaldehyde resin adhesive (UF). Flexural strength is an index that decides whether one material can be reused or the number of times that this material can be recycled, and the modulus of rupture (MOR) and the modulus of elasticity (MOE) can be a reference to judge flexural strength. The results show that the size of PF particles affects the flexural strength of the composites. The change in internal bond (IB) strength is likely to be flexural strength. But, in a contained range, the particles are smaller and the IB strength is higher. Aiming to characterize the combustion behavior of materials, Zhang and coworkers introduced the limiting oxygen index (LOI). It is generally considered that the value of LOI less than 22% belongs to flammable materials, and the value between 22–27% belongs to combustible materials. A value greater than 27% belongs to refractory materials [42,46]. Without PF particles, the panel is flammable and the LOI value is about 26%, and after adding PF particles, the LOI value rises to 37%, which is hard to burn. In addition to measuring the flame-retardant properties of composite materials by LOI, the author also carried out the CONE test. The CONE test is also an effective method to measure the flame retardancy of materials [47]. The test results are similar to LOI. The subsequent morphology of the sample can also prove the role of the filler (Figure 8b). Figure 8c shows the residual carbon of WB-PF composites with different addition levels of 80–120 mesh particles. This can intuitively judge the flame-retardant properties of the material.

In addition to using the flame retardancy of phenolic resin to prepare fire retardant materials, others have also proposed that it be used to prepare antioxidant materials. Gröning et al. proposed the idea to take advantage of waste phenolic resins as antioxidative fillers in other materials like polypropylene and polyamide-6 [48]. This idea is based on phenol–formaldehyde thermosetting resin containing a large amount of hindered phenolic sites when cured in a single uniform molecule. Hindered phenols are known to possess antioxidative properties [49] because of their hindered phenol structures like CH3- or -CH2-. Blocked phenolic structures are often found in synthetic antioxidants and play an important role in the antioxidant process of many natural polymers. The entire recycling process is shown in Figure 8d. Prior to mixing, the phenolic resin waste was cured in an air oven at a temperature set at 200 °C for two hours. The cured phenolic waste was then ground and a 5 mm diameter screen was used to obtain 4–6 mm fibers. PP and PA6 composites were prepared using a twin-screw extruder. In general, the mechanical properties of composites will decrease relative to those of raw materials. A similar situation also happened in this research. However, some experiments have shown that adding compatibilizers to composites can improve the mechanical properties of composites [50].

From Figure 8e, researchers found that the PP/prepreg composite is stable significantly longer time than the unstabilized base PP. The unstabilized base PP starts oxidizing as soon as the atmosphere changes to oxygen from an anoxic environment. On the contrary, the PP/prepreg composite started oxidizing after about 20 min, and this phenomenon verified the anti-oxygenations of waste phenolic resins fillers. At the same time, even though the PP/prepreg composite is better than the base material, the oxidation induction time was only 1/3 of the reference material. The antioxidant properties are still unsatisfactory. In fact, this result is not objective, because blocked phenols in commercial materials are often used in combination with secondary antioxidants such as phosphite or sulfur. And, if PP/prepreg composites contain phosphite or sulfur antioxidants, the OIT of prepreg composites can be additionally increased due to the synergistic effect of secondary antioxidants with phenolic prepreg.

Researchers also studied the mechanical properties during long-term accelerated aging. The result verified the anti-oxygenation of phenolic fillers, too. The result of impact measurements showed the impact strength of the base material was reduced to 30% of the initial result after 100 h. However, for the PP/prepreg composite material, the impact strength did not decrease until 500 h, while the first 500 h is basically unaffected, and the impact strength decreases to 70% of the original after 1000 h. Flexural testing showed that the PP/prepreg and the reference PP/glass fiber composites were oxidized at approximately the same rate during the first 500 h of accelerated aging, whereas the unstabilized base material was oxidized significantly faster. Then, the base material was changed to PA6, and the two tests were still carried out. The results were roughly the same as those of PP. The anti-oxidation performance of the base material was still the lowest, and the anti-oxidation performance of the prepreg material was better than that of the base material. The test results of both materials all proved the antioxidant effect of phenolic resin filler.

When people think about recycling phenolic resin, landfills are always the first method that comes to us. However, natural degradation has many negative effects. Leaving aside that the chemical substance in the degradation product may destroy the natural environment, this method has a huge time limitation. It takes about five centuries, or even longer, to degrade only one thermoplastic plastic bag. This means that thermosetting plastics will take longer to break down. Over time, the total quantity of buried waste plastic will keep increasing, and this is inevitable until the first plastic to be buried has been degraded completely after several centuries. Except for landfills, mechanical recycling is another method that should be widely applied. This kind of recovery method is usually related to composite materials because most of these are recycled into powder and as fillers or reinforcers to add to other materials. The biggest difference between fiber packing and powder packing is that fiber packing is mainly used as a skeleton to bear stress and load, so the use of fiber packing products generally has better mechanical properties. The research of Mikael Gröning [48] shows us a different perspective that takes advantage of the chemical properties. They are attracted by oxidation resistance and synthesize a kind of composite material with waste phenolic resin to enhance antioxidant properties.

When it comes to mechanical recycling, there are still many problems and challenges to be solved: (1) products prepared by mechanical recycling always have a decline in physical properties compared with raw materials [28]; (2) the process of mechanical recycling is long, and most mechanical recycling methods are to grind waste materials and then put them into manufacturing; (3) phenolic resin contains a large amount of free phenol and free formaldehyde, which may be released during mechanical recovery [48]; and (4) some wastes are not suitable for mechanical recycling because their special properties may even adversely affect the mechanical properties of recycled products [30]. At the same time, we also summarize some improvement methods for mechanical recycling: (1) by constantly adjusting the size of the filling or changing the composition of the composite material, some physical properties of the composite material may be improved; (2) using the chemical properties of phenolic resin to prepare some special materials seems to offer a new direction [39,48]; (3) the free substances emitted during the recovery process can be collected using a scavenger; and (4) perhaps we can think about this mechanical recycling problem from a broader perspective, as phenolic resins belong to the general category of thermosetting resins, and there are many materials under the same category of thermosetting resins, so perhaps we can use the recycling methods of these other thermosetting resins to recycle phenolic resins. Here, the author will not add too much detail and only presents a table to roughly list the materials of similar recycling methods (Table 1).

A large number of references in Table 1 indicate the theoretical feasibility of mechanical recovery of waste phenolic resin. The products obtained from mechanical recycling are used for various purposes, but due to the decline in mechanical properties, they are mainly used as building materials. Asphalt, as a building material with excellent durability and load-bearing capacity, is widely used for paving roads [58]. Some researchers believe that waste plastics and rubber can not only replace the aggregate in the asphalt, but also improve the performance of the asphalt [59]. Although the percentage of replacement is small, less than one percent, because the production and consumption of asphalt is so large (1.5 trillion tons), even millions of tons of plastic waste can be fully used [60]. Concrete is the most widely used building material in the world, with an annual output of 3 tons per capita. Researchers are trying to use materials such as plastic or rubber to replace the aggregate in concrete in order to reduce carbon dioxide emissions from concrete production and use. There have been many studies of the massive replacement of sand in concrete [61,62], saving about 820 million tons of sand per year. Therefore, plastic waste through mechanical recycling can be completely used in these building materials, even if the production of resin is very large, it can still be successfully recycled and put into practical use again.

In this section, it is necessary to summarize the properties of the products obtained by several recovery methods. Mechanical grinding and recycling are the easiest ways to treat phenolic resin, but this method is always accompanied by a decline in mechanical properties, such as tensile strength and impact strength. So, researchers have conducted a lot of research on how to improve the mechanical properties of recycled products as much as possible. Finally, it was determined that the particle size of phenolic resin powder was an important factor. With the increase in particle size, the tensile strength and impact strength of the material decrease. Only by reducing the particle size of the powder can the composite material with excellent properties be obtained. In the preparation of special materials, the mechanical properties of the material are no longer very important, and the special properties are the focus of attention on the recovered products. In the production of the flame-retardant board, the mechanical properties of the recovered products are not discussed in detail, but the flame-retardant properties of the products are described in large quantities and the flame-retardant properties of this product increase with the content of phenolic resin particles. Similarly, when preparing antioxidant materials, antioxidant properties are discussed in detail.

## 3. Chemical Recycling

Chemical recovery in the general sense involves depolymerizing the product using various methods, and this traditional idea has unique advantages. However, chemical conversion of phenolic resins into other materials can also be a method of chemical recovery. Most plastic waste can be upcycled into carbon materials [63,64,65]. There have been many studies that have successfully converted some thermoplastics (like PET [66,67,68,69], PVC [70], PE [71] and PS [72]) into valued chemicals or functional materials. As a result, researchers have also succeeded in converting PF into carbon materials. The types and forms of carbon materials also vary. This section will begin with a description and discussion of some of the depolymerization methods for phenolic resins. After depolymerization, the conversion of phenolic resins to other carbon materials will be explored in detail.

### 3.1. Depolymerization

**Figure 9:**
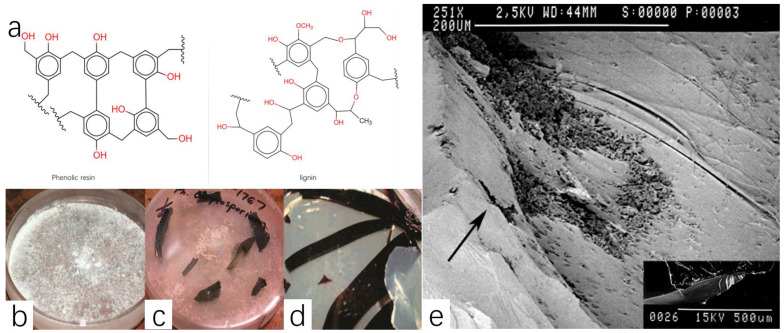
(**a**) Two-dimensional structure of phenolic resin and lignin; (**b**) Pc1 grown alone on 1.5% malt agar for 13 days (control). (**c**) 13-day-old culture of Pc1 grown on 1.5% with PR embedded for 3 days. (**d**) PR embedded in 1.5% malt agar alone for 13 days (control); (**e**) surface morphology of the sample under scanning electron microscope (The arrows are pointing to rough surfaces). Reproduced with permission from ref. [74]. Copyright 2006 ACS.

In addition to landfills and mechanical recovery, there are many recycling methods for phenolic resin. The use of chemical methods to recycle plastic waste also has great potential, which is called chemical recycling. The essence of chemical recycling is the conversion of plastic waste into smaller molecules, which can be purified and continue to act as raw materials to prepare plastic products [73]. This is also a green and sustainable recycling method, as it actually reduces the need for raw materials. Traditional chemical recovery methods have not been introduced too much, so here we present two methods with the characteristics of biodegradation and supercritical fluid degradation. These two methods can be summarized as depolymerization because both methods degrade the phenolic plastic and produce oligomers. Sometimes the raw materials for phenolic resin can even be obtained directly. The phenolic resin was considered unavailable to biodegrade previously, and, as a result, landfills and mechanical recovery are the main methods. Some enterprises and laboratories come up with several chemical methods to handle waste PF, but the cost is too high, and it is possible to produce extra waste. As a result, landfills and mechanical recovery and chemical recovery methods are not very suitable in terms of cost and environmental considerations. Therefore, biological recovery seems more attractive after thinking about multiple factors. Thus, Barr et al. discussing a white-rot fungus [74] draws peoples’ attention. White-rot fungus is a sort of wood-degrading fungi that can secrete a kind of enzyme called ligninases after lengthy evolution, and these ligninases can degrade lignin [75]. It has been shown in past experiments that ligninases can decompose a range of chemical pollutants, such as pyrenes, PCBs, dioxins and others. After many experiments and conjectures, the mechanism by which this enzyme can decompose organic pollutants is associated with its free-radical nature [76]. So many chemical pollutants have been proved that ligninases can play a role in their degradation [75]. Some articles have summarized the biodegradation of plastics, but have not mentioned the biodegradation of phenolic resins [77]. Many researchers have also successfully demonstrated the positive effect of ligninases on the degradation of some plastic products obtained by copolymerization with lignin. In terms of lignin–styrene polymer, it has a similar structure to phenolic resins (Figure 9a), and some chemists assume that phenolic resin can also be degraded by ligninases. In two other experiments, Milstein et al. [78] and Chen et al. [79] showed that three kinds of white-rot basidiomycetes, *P. chrysosporium*, *Pleurotus ostreatus* and *Trametes versicolor*, were able to biodegrade lignin–styrene copolymerization products.

In the research of D. P. Barr [74], he tested dozens of fungi to discover which is equipped to degrade phenolic resin. As a result, these enzymes, only one enzyme called *P. chrysosporium* had the potential to degrade phenolic resins (PRs). Researchers carried out some experiments to test which fungus can degrade phenolic resin. In the first experiment, scientists grew different fungi on malt agar medium, and then PR was embedded in the culture dish. After several days, observe whether the surface of PR chunks was changed or degraded. The result showed that in the culture dish where the *P. chrysosporium* was grown with PF chunks, scientists observed one kind of chromatic transformation after three days (Figure 9c). The surface of the PR chunks in the Petri dish gradually turned pink. However, there was no color change in the other two Petri dishes that served as controls, one without PR chunks (Figure 9b), and the other dish had other fungi (Figure 9d).

Researchers suggest that this phenomenon indicates that this enzyme can biodegrade polymer because the phenolic resin is a pink water-soluble substance before the polymerization. This reappeared pink substance can further decompose into phenol and formaldehyde. What needs illustration is this phenomenon appearing in the culture dish of *P. chrysosporium* after only about two days. On the contrary, in other culture dishes, this chromatic transformation did not appear, even if they extended the reaction time to two weeks later. A final piece of convincing evidence came from scanning electron microscopy of the sample. The mycelia growing on the surface of the sample hindered the observation of the sample and could not confirm whether the mycelia penetrated the polymer. After the sample was cleaned with ethanol, the surface of the sample was observed using a scanning electron microscope. The sample containing *P. chrysosporium* and PR appeared with a pockmarked surface and jagged-edged holes. Meanwhile, the sample without *P. chrysosporium* had a smooth surface. This comparison shows that the fungus does degrade the resin (Figure 9e). Phenolic resin can be biodegraded, and potentially incorporated into large-scale PR recycling processes in a short time. This enzyme could offer a new way to handle wood–polymer composites. Although biological recycling is the best choice for the environment, it is challenging and progress is slow. Despite its inefficiency and need for a specific environment to maintain fungus activity, it is a valuable method requiring further study.

**Figure 10:**
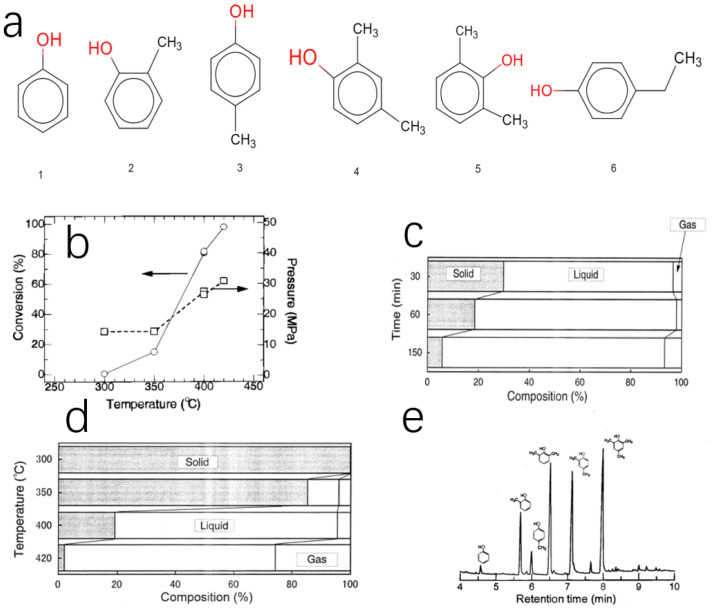
(**a**) Six major decomposition products; (**b**) reaction temperature dependence of conversion based on the solid weights before and after the reaction in supercritical methanol (The arrow points to the kind of property that corresponds to the curve); (**c**) effect of reaction temperature on product composition. The reaction time was fixed at 60 min; (**d**) effect of reaction time on product composition. The reaction temperature was fixed at 400 °C; (**e**) typical example of a GC-MS chromatogram. Reproduced with permission from ref. [82]. Copyright 2000 ACS.

In recent years, supercritical fluid has become a hot topic again [80]. Supercritical fluid refers to the fluid above the critical temperature and pressure. Since there is no obvious gas–liquid interface, the specific physical state cannot be clarified. Because the supercritical fluid is in the supercritical state, it is very sensitive to changes in temperature and pressure and has very unique physical properties. It has a density close to liquid, a viscosity close to gas and a high diffusion coefficient, so it has a strong solubility and good flow and transfer performance. Supercritical fluid is applied in various fields of production and life, such as energy saving, extraction of natural products, polymerization reaction, production of ultrafine powder and fiber, spray and coating, catalytic process and supercritical chromatography to obtain certain characteristics of products, called supercritical fluid technology. So, some people used supercritical fluids to recover phenolic resin. For example, Hideyuki Tagaya and coworkers carried out an experiment to recover phenolic resin into monomers in supercritical water (SCW) [81]. They explored the reaction mechanism and successfully obtained the decomposed monomer. In the Figure 10a, the hydroxyl group is highlighted in red so that the reader can clearly see that all six products are phenols and their homologues. After that, they discussed the factors affecting the reaction and compared it with the decomposition of other substances. There are many other people who have a similar idea and put it into action [82]. Some scientists use supercritical methanol to recover phenolic resin. Substitutions of supercritical methanol for supercritical water may be more reasonable. On one hand, the stagnation temperature and critical pressure of methanol are lower than water, resulting in the operating conditions being more relaxed. On the other hand, due to the boiling point of methanol being lower than that of water, in the phase of distillation or other separation step, it is easier to separate product from solvent.

The experiment involves mixing phenolic resin with methanol in an autoclave, which discharges air to prevent interference. The vessel is heated in an electric furnace, with a temperature controller for accurate temperature adjustments. The temperature ranges from 300 °C to 420 °C for up to 150 min. After heating, the vessel is cooled to room temperature. The solid and liquid products are weighed and the gas product composition is calculated. The solid product composition is measured using traditional combustion techniques, while the liquid product composition is measured using GC-MS equipment.

During the heating phase, the pressure increased with the heating (Figure 10b). In order to find out the reason that this phenomenon happened, a control experiment was set up without phenolic resin. The result showed that the change of pressure has no relation with phenolic resin, and the phenomenon is related to the thermal expansion of methanol fluid. The reaction conditions employed in the present study exceeded the critical point of methanol, so the methanol became the supercritical methanol. When the temperature was up to 350 °C, the percent conversion started to increase, even up to 98% when the temperature was 420 °C. Under the circumstance of keeping the temperature constant, studying the relation between reaction time and conversion. The result indicated that the conversion increased with the extension of time at the same temperature. A picture showed the product distribution of the reactions with different reaction times. As shown in Figure 10c, with the extension of time, the percent of the liquid is growing, and the gas part does not change too much. In general, the effect of extension of time or increasing temperature is similar (Figure 10d). But, lengthening the reaction time can obtain more liquid parts, so choosing to increase the reaction time is better in this experiment. The common gas chromatographic technique is not suitable for the measurement of the composition of this liquid. Because liquid products may contain some oligomer of phenolic resin. The liquid product was composed of mainly six phenol substitutes, that is, phenol, o- and p-methyl phenyl, 2,6- and 2,4-dimethyphenols and 2,4,6-trimethylphenol (Figure 10e).

In fact, reactions of phenol with methanol under high temperatures and high-pressure conditions have been known to cause methylation. This suggests a possibility of methylation of phenolic nuclei through the action of supercritical methanol. It is interesting to note there is carbonization took place in this phase, mainly shown in Table 2. With the reaction time extended, the composition of C is increasing, while the composition of O is declining.

This experiment demonstrates that supercritical methanol can recycle phenolic resin, and obtain liquid products to contain phenolic substances and oligomers of phenolic resin. Extending reaction time or elevating temperature could increase the conversion of the reaction, but in order to gain more liquid product, the former method to increase conversion should be chosen. Supercritical fluid can dissociate polymer materials well due to its special properties, but whether it is supercritical water, supercritical methanol or other solvents, there are some problems that cannot be avoided, such as cost. To obtain a material to a supercritical state, very high pressure and temperature are required, large-scale industrialization is unlikely and the high-temperature and high-pressure experimental conditions are dangerous. These issues still need to be optimized in subsequent studies. There have been many studies on the chemical recovery of phenolic resins, and Table 3 summarizes the recovery of various types of phenolic resins.

### 3.2. Conversion into Aromatic Amines

**Figure 11:**
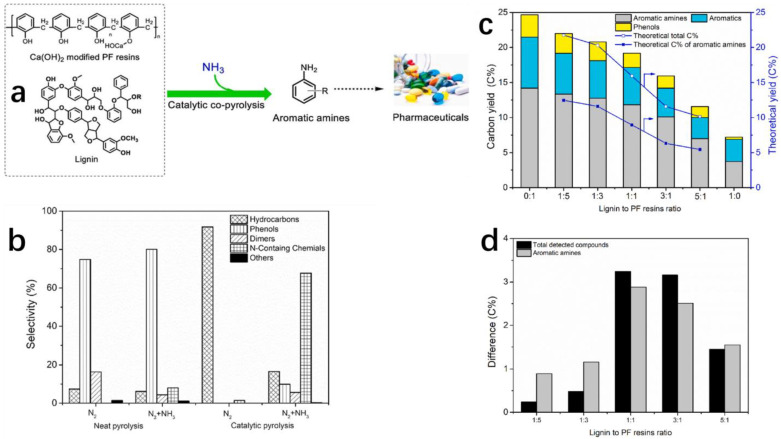
(**a**) Summary of preparation of aromatic amines by co-pyrolysis of lignin and modified phenolic resin; (**b**) pyrolysis products from PF resins under different atmospheres selectivity of chemicals in pyrolysis oil; (**c**) producing aromatic amines; (**d**) differences of theoretical and actual yields. Reproduced with permission from ref. [90]. Copyright 2020 Elsevier.

Aromatic amines refer to an amine having an aromatic substituent, namely -NH_2_, -NH or groups containing nitrogen, which are linked to aromatic hydrocarbons [88,89]. The structure of aromatic hydrocarbons usually contains one or more benzene rings, that is, the nitrogen atoms are directly connected with the carbon atoms of the benzene ring by chemical bonds, such as aniline, methyl anilines and dimethyl anilines. Industrially, they are produced by reducing aromatic nitro compounds, a commonly used reducing agent is composed of metal and acid, metal can be iron, zinc or tin, and acid can be hydrochloric acid, acetic acid and sulfuric acid. The concern is that the disadvantages seem hard to address with amounts of corrosive acid and metal catalysts such as, for example, high cost, the emission of acid wastewater and operational hazards through the production process. Therefore, Lujiang Xu and co-workers developed an efficient and safe method for directly producing aromatic amines from complex plastic waste [90]. From an environmental point of view, this method is definitely more environmentally friendly than the traditional method of producing aromatic amines, with neither the discharge of strong acid wastewater nor the need for too many metal catalysts. The aromatic amines prepared by this method are re-put into the chemical industry, forming a cycle that produces less waste (Figure 11a).

Catalytic pyrolysis has been recognized as a promising utilization method that recycles biomass and waste phenolic resins into valuable chemicals. For instance, phenolic compounds had been prepared successfully by pyrolysis of phenolic resin and lignin [91]. In recent years, nitrogen-containing compounds have also been shown to be produced from biomass and waste plastics [92,93]. However, the challenge is that the yield of aromatic amines from lignin was still low while the catalyst was applied [94]. In short, the co-pyrolysis of biomass and waste plastics was efficient in valorizing both valuable chemicals and high-quality biofuels. Synergetic interactions between lignin and PF resins might exist during co-pyrolysis to influence the yield of aromatic amines. Xu et al. [90] conducted experiments to verify the high-yield production of aromatic amines by controlling pyrolysis conditions at first. Then, they studied the synergetic interactions after adding lignin to determine whether they truly influence productivity.

Before the pyrolysis of the resin, it is necessary to modify the resin with Ca(OH)_2_. The modified resin can better promote the generation of phenol and avoid carbonization in the pyrolysis process [95]. In pure pyrolysis, the yield of pyrolytic oil is higher, and the yield of phenolic compounds occupies an absolute position no matter if it is in a pure nitrogen environment or a mixed environment of nitrogen and ammonia (Figure 11b). However, catalytic pyrolysis shows high selectivity, and almost only hydrocarbons are produced in a pure nitrogen environment. In the mixed environment of nitrogen and ammonia, nitrogen-containing compounds become the main products, accounting for about 70%. Therefore, ammonia played the role of both a reactant and a carrier gas in the pyrolysis of PF resins to aromatic amines. These results showed that ammonia with pyrolysis technology has the potential to be an affordable approach to recycling PF resins.

In order to figure out the effect of the catalyst, commercial HZSM-5-3 zeolite was added to the process and a remarkable change took place. In pure N_2_, the main product was changed to hydrocarbons from phenolic compounds, while in the mixture gas, the main product was changed to N-containing chemicals and aromatic amines. Different ratios of lignin and PF resins were added to investigate the influence of ingredient proportions. On the whole, the carbon yields of detected pyrolysis compounds and aromatic amines decreased with the mixing ratio of lignin to PF resins increasing. In addition, by comparing the theoretical yield and actual yield of aromatic amines, researchers drew a conclusion that adding PF resins in lignin significantly promoted aromatic amines production. After considering various conditions, we chose 1:1 as the best mixing rate of lignin and PF resins for producing aromatic amines (Figure 11c). When the resin and lignin were mixed for 1:1 co-pyrolysis, the results of the pyrolysis were surprising. The yield of simple phenolic compounds was nearly 10% higher than that of lignin alone, and 5% higher than the theoretical yield (Figure 11d). This strongly proves that the co-pyrolysis of resin and lignin contributes to the generation of aromatic amines. Exploring a deeper cause, the researchers found that pyrolytic phenolic resins produce methylene, which they believe acts as a hydrogen donor to convert phenol into aromatic amines [96,97].

### 3.3. Recycling to Carbon-Based Functional Materials

It is a good way to depolymerize phenolic resin into monomers or oligomers blindly, but direct conversion to functional materials may be better than depolymerization in terms of cost and environmental protection. Phenolic resin can be converted into many functional materials, which can greatly reduce the preparation cost of other functional materials, realize waste utilization and prevent the pollution of waste plastics. During the past few years, carbon-based materials, such as carbon fibers, heteroatom-doped carbon, carbon nanotubes, graphene and so on, have been demonstrated to be versatile functional materials for many applications [98,99,100,101,102,103,104,105,106]. Considering the increasing demand for carbon precursors in the market, converting waste plastics or biomass into carbon-based materials may be a good choice in terms of reserving non-renewable resources, environmental protection and economics [107,108,109,110]. Yoo et al. reported the conversion of phenolic formaldehyde resin into carbon xerogels, which demonstrates excellent electrochemical performance as an electrode material for organic EDLCs [111]. Veselov et al. studied the factors affecting the carbon xerogels and explored the effect of adding metal elements on the carbon xerogels [112]. By co-calcining nano-CaCO_3_ and phenolic resin followed by an etching process, Liu et al. prepared nanoporous carbon. Owing to the introduction of nano-CaCO_3_, which is expected to act as a hard template in forming nanoporous carbon, the obtained carbon produces pore distribution concentrated around 50 nm with a BET surface area of 543 m^2^/g. For practice application, the nanoporous carbon delivers a reversible capacity of 260 mAh/g at a current density of 200 mA/g with good rate capability (145 mAh/g at 2000 mA/g) and cycling performance (retaining 92.4% of the initial reversible capacity after 100 cycles) when used as an anode material for lithium-ion batteries [113].

**Figure 12:**
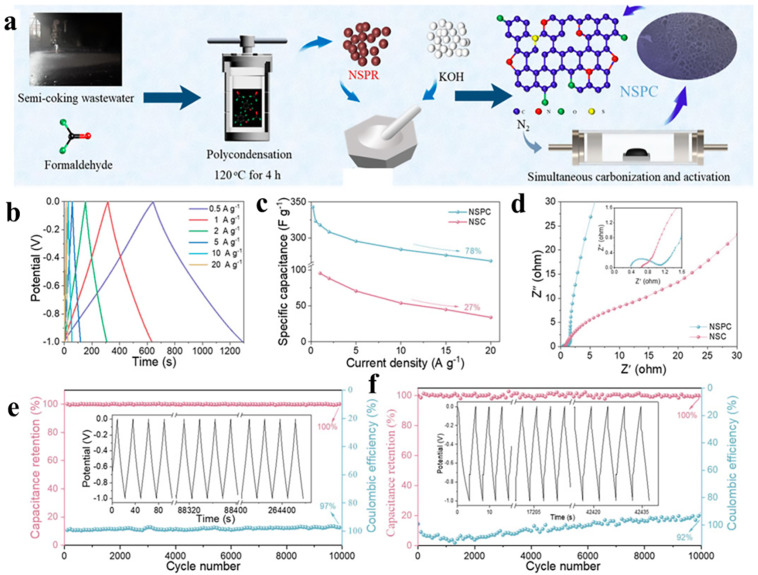
(**a**) Synthesis of NSPC from semi-coking wastewater and formaldehyde; (**b**) GCD curve of NSPC at various current densities; (**c**) current density dependent specific capacitance of NSPC and NSC; (**d**) EIS spectra of NSPC and NSC. Cycling stability and Coulombic efficiency of NSPC (**e**) and NSC (**f**). Reproduced from ref. [114] with permission. Copyright 2022 published by Frontiers under a CC-BY license.

Besides directly converting waste PF resin into carbon, “waste” PF resin can also be synthesized using waste phenolic raw materials and then converted into carbons since contamination of phenolic compounds has devastating effects on the environment. For example, Yan et al. [114] synthesized N-S-doped phenolic resin (NSPR) from phenols, N and S groups in semi-coking wastewater, and formaldehyde, which can be further converted into N-S-doped porous carbon (NSPC) through KOH-activated carbonization (Figure 12a). For comparison, NSPC-derived carbon without KOH activation (NSC) is also prepared. They found that NSPC exhibits a superior porous structure over NSC. The specific surface area of NSPC reaches up to 2523 m^2^/g with a numerous total pore volume of 1.30 cm^3^/g. These texture features lead to facilitated mass/charge transformation. In addition, the heteroatom doping level is very high with 0.76 at% N and 0.914 at% S. Furthermore, the electrochemical performance of NSPC and NSC are also investigated by comparison. As revealed in Figure 12b–f, the NSPC exhibits better electrochemical performance than NSC in view of specific capacitance, rate capability as well as cycling stability [114].

Activated carbon (AC) is a material prepared from carbon-containing substances, such as wood, coal and petroleum coke. Because of the developed pore structure, large specific area and abundant surface chemical groups, AC has strong adsorption capacity. AC is used to remove pollution or concentrate small compounds and other applications [115,116,117,118,119]. There are various methods for preparing activated carbon, and a large number of studies have explained the mechanism of activation and carbonization [120]. In actual life, converting waste into activated carbon is more cost-effective and practical. Industrial waste recycling to prepare activated carbon has been successful and widely used [121,122]. Considering the high carbon content of phenolic resin, many researchers have successfully prepared activated carbon by pyrolysis of waste phenolic resin. In other words, the use of waste phenolic resin to prepare activated carbon is completely feasible. Activated carbon has a very broad market and prospects, and has been widely used in water purification, energy storage, as a catalyst and in other fields [123]. From the perspective of water purification alone, the demand for activated carbon is very large. At present, 70% of the world’s fresh water is used for irrigation and food production, and this figure will rise to 90% as the global population increases in the future. In developing countries, environmental protection is neglected in the pursuit of economic development, and about 70% of industrial wastewater is discharged directly into rivers and oceans. With the increasing demand for fresh water and the decrease in fresh water resources, a global fresh water crisis is likely to occur in the coming decades [124]. Therefore, the purification of sewage has become more urgent and important. In a large number of water purification methods, the use of activated carbon is simple, low-cost and demonstrates high efficiency, and these advantages make the demand and application of activated carbon very broad. A large amount of phenolic resin waste can be completely converted into activated carbon to achieve value-added recovery. Only in 2021 did China’s activated carbon demand reach 0.73 million tons, and, according to the future development trend forecast, by 2028, China’s activated carbon market size can reach CNY 13.6 billion.

**Figure 13:**
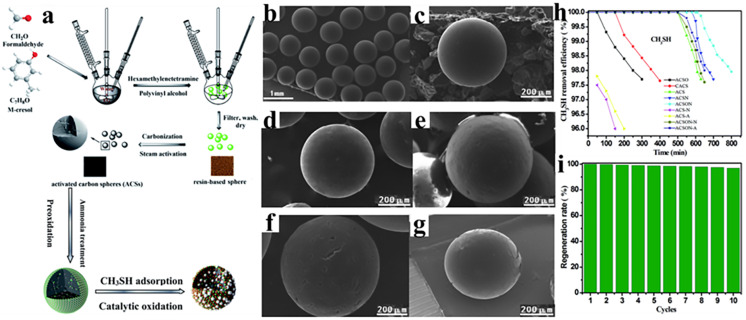
(**a**) Schematic illustration for synthesis of ACS and their adsorption/catalytic oxidation of methyl mercaptan. SEM images of (**b**,**c**) ACS, (**d**) ACSO, (**e**) ACSN, (**f**) ACSON and (**g**) CACS; (**h**) the CH_3_SH removal efficiency of all the samples; (**i**) regeneration cycles of ACSON for CH_3_SH removal. Reproduced from ref. [125] with permission. Copyright 2020 published by the Royal Society of Chemistry under a CC-BY license.

In another work, Zhang et al. [125] provided an experimental reference to convert PF resin into carbon, which is capable of catalyzing the oxidation of methyl mercaptan, as illustrated in Figure 13a. In their work, the PF resin is synthesized using isopropylphenol and formaldehyde as raw materials with a suspension polymerization technique. The obtained PF resin can be further converted into porous carbon through steam activation. In order to tune the surface and pore structures of the final carbon products, the effects of air oxidation and ammonia solution heat treatment are studied. For convenience, the activated carbon spheres are named ACS, whereas the oxidized carbons are termed ACSO. Ammonia solution-rerated samples are marked as ACSN, and both air oxidation and ammonia solution-treated samples are marked as ACSON. As revealed by Figure 13b–g, these carbon products produce spherical shapes with uniform sizes. N_2_ adsorption isotherm measurement indicates that the optimal sample (through direct ammonia treatment) has a high surface area value of 1710 m^2^/g and a total pore volume of 0.83 cm^3^/g. When used for adsorption/catalytic oxidation of methyl mercaptan, ACSNO offers the best removal efficiency, as evidenced by Figure 13h. This result can be assigned to the basic nitrogen-containing functional groups. Furthermore, the ACSNO reserves a CH3SH capacity of 97% after ten cycles (Figure 13i), demonstrating that the porous carbon spheres obtained by a preoxidation-assisted enriched nitrogen strategy are promising for the catalytic oxidation of CH_3_SH. The same group also demonstrated that ACS obtained by this strategy can be used as an adsorbent for CO_2_ capture, showing a good linear correlation with the micropore volume at higher pressures. Maximum CO_2_ adsorption capacities of 4.53 mmol/g and 13.62 mmol/g at 25 °C under 1 bar and 6 bar are achieved, respectively. The thermodynamic parameters reveal that CO_2_ adsorption conforms to physical adsorption mechanisms. Significantly, the prepared ACSs can be regenerated easily and the CO_2_ adsorption capacity shows no obvious decrease after multiple cycles [126]. Liu et al. [127] developed a two-stepped process to prepare N-doped carbon from commercial PF resin. Different from traditional carbonization-nitriding-activation three-step procedures, their synthesis only involves two steps, mixing phenolic resin and urea to form the nitrogen-enriched precursor and carbonization accompanied by KOH activation. The resulting N-doped carbon demonstrates excellent CO_2_ adsorption performance with a maximum CO_2_ uptake of 5.01 and 7.47 mmol/g at 25 and 0 °C under ambient pressure, respectively, as well as outstanding CO_2_/N_2_ selectivity and good dynamic CO_2_ uptake. By introducing melamine into the synthesis system of PF resin, N-doped PF resin is developed by Yang et al. [128], which can be further converted into ultra-microporous carbon materials. By varying the ratio of melamine, the nitrogen-doped content can be preciously tuned. Meanwhile, the activation strength and size of the alkali ions result in controlled pore sizes ranging at the sub-Ångström level from 5.8 to 7.7 Å with precision between 0.1 and 0.4 Å. The obtained ultramicroporous carbon exhibits the capability of recovering SF6, which is larger than conventional molecules such as CO_2_. Under optimal conditions, it shows an increase in SF6 adsorption from 0.39 to 6.55 mmol/g while the SF6/N_2_ selectivity reached as high as 78.8.

Kuan and coworkers studied a novel method to prepare AC in order to remove ammonium in the water [129]: the conversion of phenolic resin waste to activated carbon with a microwave-catalyzed reaction. The process of converting phenolic resin to activated carbon is roughly divided into two steps: activation and carbonization. Carbonization is the process by which organic materials are heated without air to reduce their non-carbon content. Oxidation activation can further remove the residual volatile substances, generate new and expand the original pores, improve the micropore structure and increase the activity. The carbonization process of activated carbon is generally carried out in an electric heating (EH) furnace. What makes their experiment different is that it uses microwave heating instead of traditional electric heating, which has many advantages, such as higher heat transfer efficiency, lower heating temperature, energy savings, the heating part can be selected, it has a fast start, there is easy operation of equipment and so on [130,131,132,133].

**Figure 14:**
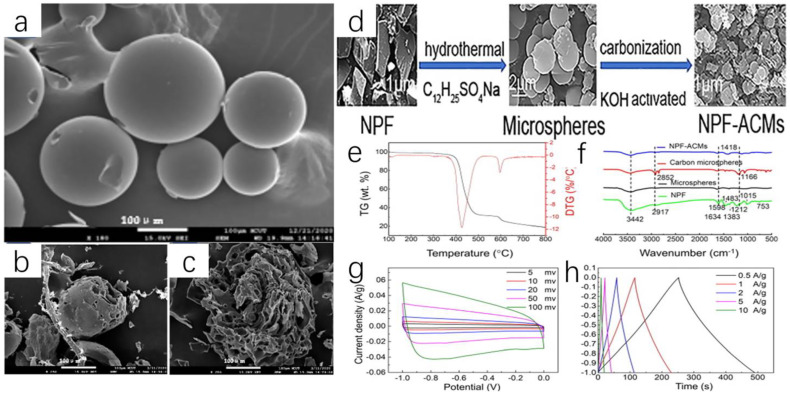
(**a**) Scanning electron microscope view of the topography of raw phenolic resin waste; (**b**) activated carbon (AC) impregnated in phosphoric acid and subjected to microwaves (ACP-MW); (**c**) AC activated using KOH and subjected to microwaves (ACK-MW). Reproduced with permission from ref. [129]. Copyright 2021, published under a CC-BY license; (**d**) schematic for the preparation procedure of NPF-ACMs; (**e**) TGA curves of NPF-ACMs; (**f**) FTIR spectra images of NPF, microspheres, carbon microspheres, NPF-ACMs; (**g**) cyclic voltammograms of NPF-ACMs; (**h**) typical charge–discharge curves of NPF-ACMs. Reference with permission from ref. [134]. Copyright 2020 Elsevier.

In the activation process, they selected two activators, KOH and H_3_PO_4_, to activate the raw materials. Both activators have activation functions but are different from the dielectric. Field emission scanning electron microscopy (SEM) was used to observe the surface of the original phenolic resin and the product of two different activation and carbonization modes. It can be seen that the surface of phenolic resin is very smooth, with almost no porous structure (Figure 14a). The surface of activated carbon products with different combinations seems to reflect the influence of different activators and heating methods. The products activated by KOH have more porous structures and deeper pores on the surface of the products (Figure 14b,c). This kind of AC has a higher specific surface area than other AC, the reason for which can be attributed to microwave radiation and activators. Under the influence of these two factors, enhancing porosity and surface functional group formation, the activator was the dominant factor determining the AC porosity and surface chemical features. Because of the alkali-enhanced hydrolysis of the methylene linkages in the phenolic resin and its subsequent decomposition, the KOH activator strongly etched the resin and then developed a pronounced porous structure.

Novolac phenol formaldehyde (NPF) resins are made up of pure phenolic resins or modified phenolic resins and vegetable oils. NPF has nice oil resistivity, water-resistant properties, acid resistance and insulativity. Thus, NPF is used to bond plexiglass and the surface coating of insulating parts. However, the effluents containing NPF always contain phenol and formaldehyde. Although traditional treatments such as extraction [135], oxidation and biotechnology can address this problem, they all have drawbacks such as efficiency and cost. To further improve traditional methods, Mingjie Guan and colleagues have developed a novel treatment method using waste NPF to produce activated carbon microspheres (ACM) [134]. ACM is widely used as a battery negative electrode because of its many characteristics, like designable particle size, regular configuration and adjustable porosity. Therefore, the research of ACM on supercapacitors and lithium-ion batteries is very promising. The use of recycled materials to prepare lithium battery electrodes and other components sounds very attractive and very environmentally friendly.

After washing and drying, the NPF microspheres were carbonized at a high temperature (800 °C), and then mesoporous carbon microspheres were prepared by standing in N_2_ for one hour. Then, they were further activated by KOH at 800 °C and maintained for one hour in the nitrogen environment. In this way, NFP-ACM was prepared successfully (Figure 14d). The researchers used scanning electron microscopy (SEM) and thermogravimetric analysis (TGA) to better understand the morphology and microstructure of the NFP-ACMs microspheres and also tested the electrical properties of the microspheres for their applications in capacitors (Figure 14e–h). Through the characterization of FTIR (Figure 14f), we can simply summarize the synthesis mechanism in two steps: first, at a low temperature, the -OH transformed into C-O-C and then some C-O-C transformed into methylene bonds.

To evaluate the electrochemical behavior, after the CV measurements (Figure 14g) were completed, researchers found that NPF-ACMs were equipped with a double-layer capacitance effect, good energy storage characteristics, and fast charging–discharging performance. The shape of the CV curve is nearly rectangular, and there is no obvious redox peak, indicating that the precursor has a double capacitance effect. This double-layer effect can also be seen in the current density diagram (Figure 14h). Finally, the stability of the material is tested. After 1000 cycles of testing, the results show excellent electrochemical stability.

Due to large-scale industrial production and occasional oil spills, oil and water separation has become an almost global industrial and environmental issue. There are many traditional methods of oil and water separation, which can be divided into the density difference principle separation method, air flotation method, flocculation method and adsorption method. The adsorption method is a technology used to treat oily wastewater, which also occupies a very important position. The principle is to increase the surface area of the adsorbent so that more dissolved oil and organic matter can be adsorbed on the adsorbent, so as to achieve the effect of oil–water separation. Thus, an efficient adsorbent can be designed for better separation. J-y Qu and co-workers invent a novel approach to the synthesis of carbon foams as an adsorbent by recycling phenolic resin waste [136].

**Figure 15:**
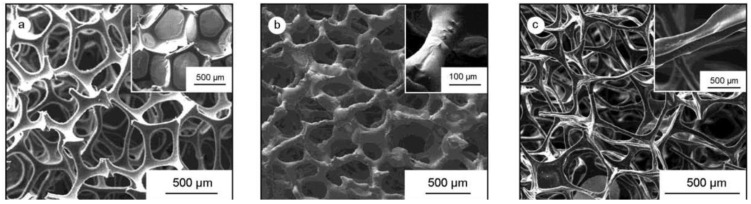
SEM images of (**a**) PU*, inset is the pristine PU template, (**b**) the PU*/LPF composite after impregnating PU* template with LPF, (**c**) the obtained carbon foam after carbonization of the PU*/LPF composite at 950 °C. Reproduced with permission from ref. [136]. Copyright 2017 Elsevier.

The carbon foams were prepared using lignin–phenol–formaldehyde (LPF) resin as the carbon source and polyurethane (PU) foam as the template (Figure 15a). The PU foam was immersed in NaOH solution for 1 h at the temperature of 50 °C to obtain a high open-cell template (Figure 15b). Then, the resin is mixed with PU foam and subjected to thermal polymerization to obtain the PU/LPF composite material (Figure 15c). Compared with the original PU template, the PU template treated with sodium hydroxide has an open pore structure and larger open pores. After soaking the PU template with LPF, a pore structure similar to that of the PU template treated with NaOH was observed in the composite. After pyrolysis, the foam ligament of the material shrinks by about half, and the bulk density of the foam decreases.

Carbon foam, a waste resin, absorbs oily materials or organic solvents, increasing its weight by 10–40 times due to its low density and high porosity. Its absorption capacity is twice that of PU templates, making it easier to recycle and reuse. Carbon foam is also more environmentally friendly and cheaper than petroleum derivatives and can be used to produce carbon value-added materials. Green recovery of phenolic resin has been studied due to its large carbon content. Currently, the trend is to use phenolic resin to prepare activated carbon or electrode materials, which can adsorb pollutants in water and the environment, and reduce environmental harm by using carbon materials instead of heavy metals as electrodes.

The section on chemical recycling begins with a description of some common depolymerization methods to obtain some chemical substances. Then, it starts to explore the conversion of phenolic resins to functional materials, such as aromatic amines and carbon materials. The section on recycling phenolic resin into carbon materials is divided according to the uses and forms of various carbon materials. The first is the electrode material, which includes carbon xerogels, nanoporous carbon and doped porous carbon. As an electrode material, the carbon dry gel converted from phenolic resin showed better physical structure and electrochemical properties than the nanoporous carbon obtained by the other method. The average pore diameter of the best carbon dry gel samples is only 1.8 nm, while the average pore diameter of the nanoporous carbon is concentrated around 50 nm. Under the same measurement method (calculated by BET plot), the specific surface area of carbon dry gel (1373.3 m^2^/g) is about twice that of nanoporous carbon (543 m^2^/g). In terms of long-term durability, the carbon dry gel electrode material still has an initial capacity of 92.2% after 2000 cycles. The N-S-doped porous carbon (NSPC) prepared by doped phenolic resin has a larger specific surface area (2523 m^2^/g) and total pore volume (1.30 cm^3^/g). The electrochemical performance of N-S-doped porous carbon (NSPC) is also the best among the three carbon electrode materials. The second conversion direction is the preparation of activated carbon from phenolic resin for decontamination and other applications. When used as an adsorbent, each activated carbon product absorbs different objects, so the performance of the product can only be judged by comparing their specific surface area and the total pore volume. The performance of several products can be compared intuitively using Table 4. The literature mentioned in this article is only a small part of a large body of literature that discusses the use of phenolic resins to prepare a wide variety of carbon materials [137,138,139].

## 4. Conclusions and Outlook

Phenolic resins (PFs), the first commercial synthetic resin, have a history of over a century and are crucial in the production of various materials and products. PFs are typically synthesized from phenol and formaldehyde under acidic or alkali conditions, but their waste has become harmful to the environment and difficult to handle. Recycling is difficult due to the cross-linked network of phenolic resins. Mechanical recycling and landfills are the two most common methods for recycling waste phenolic resin, but, compared with landfills, mechanical recycling has less pollution and higher regeneration value. Ordinary mechanical recycling is divided into two steps: grinding and reproduction. This method is very simple and practical, but it is always accompanied by a reduction in mechanical properties. Another mechanical recovery method is the simple composite of phenolic resin with other materials to prepare some functional materials, and the advantage of this method is that it can make good use of some chemical properties of phenolic resin. Chemical recovery methods can be roughly divided into two categories: one is the more traditional depolymerization wherein the principle is to use various methods to break the chemical bonds between the phenolic resin cross-linked. The second method is to use some chemical means to convert the phenolic resin into carbon materials, and the feasibility of conversion to aromatic amines is discussed. Overall, there are still many challenges in how to recycle phenolic resin in a more green manner: (1) mechanical recycling can result in degradation of the properties of the regenerated products; (2) the cycle of biological recovery is too long, and it has certain requirements for the environment; (3) some methods of depolymerization such as the recovery of resin by supercritical fluids require a lot of energy; and (4) the properties of carbon materials prepared from waste phenolic resin are unstable. In order to solve these problems, there are some schemes for reference: (1) In mechanical recovery and regeneration, the mechanical properties can be optimized by changing the compound formula. (2) The study of substances with similar structures to phenolic resins may be instructive, and the fungal biodegradation of lignin provides inspiration for the degradation of phenolic resins. Although many researchers have conducted a lot of research on the green production or recycling of phenolic resin, these problems are still not perfectly solved. It is necessary to further study and discuss more green and sustainable production and recovery of phenolic resin.

## Figures and Tables

**Table 1:** Processing methods and recycling uses of various polymer plastics.

Raw Waste Material	Formulation	Condition	Application	Ref.
Phenolic resin (PF)	Feed particle diameter of 0.43 mm, feed volume of 60 g	Rotation speed of 2820 r/min, time of 80 min	Sheet material	[35]
Fiber-reinforced polymer (FRP)	5% glass fiber reinforced plastic waste fiber	In water at 20 °C and in oven at 50 °C	Concrete material	[51]
Polyurethane (PU)	70 wt% recyclate granules, 30 wt% resin binder	-	Sandwich materials	[52]
Melamine–formaldehyde (MF)	0–60 wt% MF waste	-	Concrete material	[53]
Thermosetting plastics	The ratio of cement, sand, fly ash and plastic: 1.0:0.8:0.3:0.9	-	Lightweight concrete	[54]
Epoxy resin (EP)	the particle size of 0.45 mm, the column thickness of 2 cm	-	Separating oil–water mixtures	[55]
Bulk molding compound (BMC)	-	-	Fillers or partial Reinforcement	[56]
Glass–polyester composites	40 wt% recovered glass fibers	Mixing temperature: 200 °C for 8 min; torque level: 800 gm	New thermoplastic composites	[57]
Glass fiber-reinforced SMC	10 wt% direct weight substitution	Cured at 145 °C and 4.1 MPa for 3 min	As reinforcement in new DMCs	[29]

**Table 2:** Elemental composition of the solid products after the reactions varying the nominal reaction time (①②③ indicate samples with different reaction time). Reproduced with permission from ref. [82]. Copyright 2000 ACS.

Sample	Temperature (°C)	Nominal Time (min)	Composition (%)		
			C	H	O
Phenol resin			74.9	5.5	19.6
Solid residue ①	400	30	84.2	6.0	9.8
Solid residue ②	400	60	84.9	6.0	9.6
Solid residue ③	400	150	89.9	3.9	6.2

**Table 3:** Chemical recovery conditions and products of various typical phenolic resins.

Sample	Reaction Condition	Reaction Product	Ref.
boron phenolic resin	at 1000 °C for 10 min	H_2_, CO, CH_4_, C_2_∼C_4_ hydrocarbons	[83]
high-carbon phenolic resin	at 1000 °C for 10 min	H_2_, CO, CO_2_, CH_4_, C_2_∼C_4_ hydrocarbons	[83]
silica-containing phenolic resin	at 1000 °C for 10 min	H_2_, CO, CH_4_, C_2_∼C_4_ hydrocarbons	[83]
resole phenolic resin	-	char, volatile organic compounds (VOCs) and permanent gases	[84]
phenolic resin	Ga-modified ZSM-5 catalysts; at 800 °C	monocyclic aromatic hydrocarbons	[85]
printed circuit boards	at 400 °C; NaOH; H_2_O	phenol and some phenolic compounds	[86]
phenolic epoxy vinyl ester resin	at 160 °C; KOH/C_2_H_5_OH-H_2_O	styrene and methacrylic acid, novolac glycidyl ether	[87]

**Table 4:** Rough comparison of properties of different activated carbons.

Sample	Specific Surface Area (m^2^/g) *	Total Pore Volume (cm^3^/g)	Long-Term Adsorption/Desorption Cycling Stability	Ref.
ACSON	1710	0.83	97% after ten cycles	[126]
Ph_6_M_4_Cs11	2020	0.968	Almost unchanged after ten cycles	[128]
ACK-MW	924	0.46	-	[129]

* Surface area calculated by the BET method.
